# News and Events of the Week

**Published:** 1911-02-11

**Authors:** 


### News and Events of the Week.

A display of work will be given by the Women's Sick and Wounded Convoy Corps, on Wednesday, February 22, at 4.45—6.45 p.m., in the London Scottish Drill Hall, 59 Buckingham Gate, S.W. (by kind permission of the Commanding Officer).

The International Medical Association to assist in the Suppression of War will assemble in Congress during September, 1911, in Paris, at a date to be fixed later and which will be communicated to all those interested. Medical men desirous of attending this Congress are asked to send in their names as soon as convenient to the headquarters of the Association at the address of the President, Dr. J. A. Rivière, 25 Rue des Mathurins, Opéra, Paris.

According to our contemporary *Medical Missions at Home and Abroad* there is at present a peculiar lack of young medical men for foreign mission service. Nearly all the missionary societies want men, and the supply is not forthcoming from the schools to meet the demand. The statistical Atlas of Christian Missions published in June of last year shows that there are 641 men and 341 women medical missionaries connected with evangelical missions. These have 550 hospitals under their charge, and in 1908, in-patients numbered 164,245, out-patients 4,231,635, and 144,708 were visited in their homes. The great majority of medical missionaries work in China and India. Japan has a few, but is quickly learning to meet its own medical needs. Korea, however, offers a good field for activity, as does Africa.

According to the *Zenana*, a very real difficulty has to be faced in connection with the higher education of Indian girls. It would seem that in many cases their physique is not sufficiently robust to stand the strain of hard mental work, with the result that after obtaining their diploma they readily become a prey to disease such as consumption. In this connection Mr. Prior, one of the Government inspectors in Bombay, writes in the Report of the Fifth Quinquennial Review of the Progress of Education in India, 1902-7 : " I confess that I prefer to suspend judgment as to the benefit of secondary education for native girls in India. It appears to me that with Hindu girls, at any rate, it involves a strain which they are ill qualified to bear, and even in the case of Parsi girls I am often compelled to think that the advantages are very doubtful. Moreover in this country marriage is the almost inevitable destiny of woman, and beyond the power of conversing in English, a secondary education commonly contributes little that is of much service in later life." His opinion is shared by Miss Ashworth, an inspectress in Bombay, who points out the mental strain inevitable to teaching in a foreign language, as well as the tendency to cram inseparable from the study of books which are beyond the comprehension of the girls.

The library of the Pasteur Institute of Paris, in which already reposes the bust of the master, is to be further adorned with the busts of some of its benefactors. Those of the Czar Alexander III. one of its first subscribers, the Emperor Dom Pedro II. of Brazil, Madame Boucicaut, Madame Furtado-Heine, M. Alphonse de Rothschild, Count Lauhespin, M. Chauchard and M. Osiris are to join those of Pasteur's old collaborators, Grancher, Nocard and Chamberland. Dr. Paul Richer, of the Academy of Medicine, is responsible for the marble presentments of the two last named. Portraits of MM. Duclaux, Roux and Metchnikoff already adorn the library.

" Mayfair and Town Topics," for February 2, has an excellent coloured cartoon of Mr. A. W. Mayo Robson (" Science and Sport "). The weekly can be obtained at all bookstalls at the price of sixpence.

The following Lectures at the Central London Throat and Ear Hospital, Gray's Inn Road, W.C., will be given this week : Tuesday, February 14, at 3.45 p.m., Tracheoscopy, &c., Mr. J. Gay French ; Friday, February 17, at 3.45 p.m., Clinical Pathology, Dr. Wyatt Wingrave.

The following appointments at the Manchester Royal Infirmary on January 31 were confirmed : Mr. Donald E. Core, M.D., Ch.B. (Vict.), M.R.C.P., as Resident Medical Officer at the Infirmary; Mr. E. E. Hughes, M.B., Ch.B. (Vict.), as Resident Medical Officer at the Barnes Convalescent Hospital, Cheadle; Mr. F. E. Tylecote, M.D., Ch.B. (Vict.) M.R.C.P., as Medical Registrar at the Infirmary.

Miss Beatrice Copestake has been appointed Masseuse and Massage Instructress at the Royal National Orthopædic Hospital, Great Portland Street, and the first term of the new Masseuse School will begin on March 1 next. Full particulars may be obtained on application to the Secretary. Lectures on anatomy and physiology are to be given by the Honorary Surgeons of the Hospital, and bandaging classes by the Matron. Further details of the School are to be found in another column.

We have received a pamphlet on Plague in India, being a paper read on May 18, 1905, before the Indian Section of the Society of Arts, by Charles Creighton, M.D., and now reprinted (by permission) by the Leigh-Browne Endowment (London : George Bell & Sons. 1910. Price, 3d.) Although no reason is given in the pamphlet for the reappearance of the paper in its present form at a date so remote from that of its original publication, it makes interesting reading in view of the epidemic of plague which is now devastating whole districts in China. After enumerating those parts of the Bombay Presidency and the North-West visited by himself in a tour of three months' duration, giving statistics of mortality rates for several years past in these localities, the author proceeds to describe the measures which should be taken to stamp out the scourge. He pins his faith to the evacuation of plague spots, and the building of houses with brick and concrete in lieu of the mud huts which serve to house so many of the rural population. For him plague is the result of a ground infection, though he acknowledges that in the flea theory of plague-transmission there is very promising field of academic inquiry. He is of the opinion that the disease is strictly epidemic in that, although it recurs in some cases almost every year, it diminishes in severity or tends to do so with each fresh attack. Consequently although the mortality may not show much diminution at first, since new districts may be attacked with a consequent severe local death roll, the future of India as regards the disease is a hopeful one, especially if an organised attempt were made to improve the class of dwelling for the poorer portions of the population. It is possible that the author might modify some of his assertions were he rewriting instead of reprinting a six-year-old paper. Much good work has been done in India with regard to plague since the author's lines were penned.

